# Sometimes missing the heat: the risk of underestimating extreme heat days with daily maximum heat index approximation

**DOI:** 10.1007/s00484-025-03001-7

**Published:** 2025-08-12

**Authors:** Weixuan Rosa Xu, Keith W. Dixon, Nicole Zenes, Dennis Adams-Smith

**Affiliations:** 1 Cooperative Institute for Modeling the Earth System, Atmospheric and Oceanic Sciences Program, Princeton University, Princeton, NJ USA; 2 NOAA Geophysical Fluid Dynamics Laboratory, Princeton, NJ USA; 3 Science Applications International Corporation, Reston, VA USA; 4 University Corporation for Atmospheric Research/CPAESS, Boulder, CO USA

**Keywords:** Heat index, Multivariate indices, Heat stress, Extreme events, Climate impacts

## Abstract

The heat index (HI) metric is used in many studies linking weather conditions (observed or climate model-projected temperature and humidity) to health impacts. Because climate models often provide only daily data, a common approximation method estimates the daily maximum HI (*hismaxest*) from daily maximum temperature (*tasmax*) and minimum relative humidity (*hursmin*), assuming they coincide with the actual peak HI (*hismax24*). This study evaluates the accuracy of this approximation using hourly station-based data from NOAA’s Integrated Surface Database (ISD-Lite) and the U.S. Climate Reference Network (USCRN). Though we find that *hismaxest* either matches or slightly underestimates *hismax24* for most summer days and locations, it significantly underestimates the occurrence of extreme heat days in the hottest regions. Specifically, *hismaxest* misses over 35% of days exceeding the 95th percentile of June–July–August *hismax24* at 7 of the 37 stations examined, despite performing well in cooler, drier regions. This underestimation follows an increasing nonlinear relationship with local HI thresholds, indicating that *hismaxest* may underestimate extreme heat risks in vulnerable regions of the southern U.S. These discrepancies arise because HI is more sensitive to relative humidity in hotter regions, making *hismaxest* less reliable. Our findings quantify an often overlooked uncertainty arising from daily climate model data resolution that can be comparable to scenario and model sensitivity uncertainties. These results highlight the need for careful interpretation of climate model heat projections, and emphasize the value of archiving hourly climate model data for multivariate index calculations.

**Supplementary Information:**

The online version contains supplementary material available at 10.1007/s00484-025-03001-7.

## Introduction

Weather episodes that induce high physiological heat stress in humans lead to a greater incidence of adverse health outcomes and other impacts detrimental to usual daily activities (Sarofim et al. 2016; Ebi et al. 2021; Bell et al. 2024). When extreme heat exceeds an individual’s capacity for thermoregulation, a dangerous elevation of body core temperature can result, which in serious cases may manifest as life-threatening heat stroke (Wang et al. 2016; Epstein and Yanovich 2019). During periods of extreme heat, mortality rates have been shown to rise among vulnerable groups, such as the elderly (Bunker et al. 2016) and those with pre-existing medical conditions, including cardiovascular (Liu et al. 2022), respiratory (Anderson et al. 2013a), and kidney diseases (Liu et al. 2021). Extreme heat can also adversely impact pregnant women (Chersich et al. 2020) and labor productivity (Zhao et al. 2021; Parsons et al. 2022). Though different statistical methods yield varying estimates with non-negligible uncertainty ranges, studies have attributed several thousand premature deaths per year to heat in the U.S. alone (Shindell et al. 2020; Weinberger et al. 2020; Khatana et al. 2024). The combined influence of environmental shifts and demographic changes suggest a growing risk of heat-related health issues, spurring interdisciplinary research to better understand the connection between extreme atmospheric heat and health, to inform public health planning (Hess et al. 2023).

Thermal stress indicators (TSIs) are quantifiable metrics intended to link environmental factors to the potential risk of heat-related illnesses or discomfort. Ioannou et al. (2022) performed a systematic literature review identifying 187 TSIs that can be computed from combinations of two, three, or four types of meteorological variables (temperature, humidity, radiation and wind). Some of the TSIs are designed only for hot or cold conditions, while others apply year-round. Attempts to rate different TSIs have been made (e.g., De Freitas and Grigorieva 2017); however, as Grundstein and Vanos (2021) emphasize, no single TSI is universally applicable, given differences in underlying assumptions and the characteristics of the population of interest.

Our study examines the heat index (HI) diagnostic measure used operationally by the United States National Weather Service (NWS) in the summer (Hawkins et al. 2017). The NWS HI is a function of near surface air temperature (*tas*) and relative humidity (*hurs*) and traces its pedigree to the work of Steadman (1979), which developed a summertime apparent temperature comfort index based upon a physiological model of an idealized human in shade with a light breeze, taking into account heat transfer and clothing factors. The NWS HI is the focus of this study due to its common use in U.S. heat risk communications and its use as a diagnostic metric in peer-reviewed research across disciplines, including examinations of human physiological responses (e.g., Vecellio et al. 2022) and epidemiological studies utilizing past weather observations (e.g., Metzger et al. 2010; Wellenius et al. 2017; Vaidyanathan et al. 2019).

To date, climate models typically have not calculated HI or other TSI values directly as they run. Instead, HI can be determined after the model runs, using model-archived temperature and humidity datasets as inputs. The frequent absence of hourly or sub-hourly temperature and humidity data in many climate model outputs presents a challenge for determining model-simulated daily maximum HI. This study aims to assess the reliability of one approximation method that uses daily weather measurements to approximate daily maximum HI values. More specifically, we examine the reliability of estimating the daily maximum HI from the daily maximum temperature (*tasmax*) and the daily minimum relative humidity (*hursmin*) using the NWS HI algorithm. We refer to this estimation method as *hismaxest.* Examples of prior studies that have used the *hismaxest* approximation include the gridded meteorological observation and reanalysis-based studies of Tuholske et al. (2021) and Ahn et al. (2024), the weather station-based study of Lee et al. (2021), the seasonal climate model forecasting work of Jia et al. (2024), and several multi-decadal climate model projection studies (e.g., Fischer and Schär 2010; Pierce and Cayan 2016; Russo et al. 2017; Dahl et al. 2019; Hoang et al. 2022; Licker et al. 2022). The use of daily *tasmax* and *hursmin* when estimating a daily maximum TSI value from climate model output also has been used for humidex, another TSI that is a function of temperature and humidity (Diaconescu et al. 2023; Chow et al. 2024).

## Data and methodology

### Data

For this research, we used station-based hourly observational weather data from two primary sources. The first dataset comes from National Oceanic and Atmospheric Administration’s (NOAA) National Climatic Data Center (NCDC) Integrated Surface Database (ISD) project. ISD is a global collection of synoptic weather observations from over 100 sources. Specifically, we used the ISD-Lite product (National Centers for Environmental Information 2006), a subset of the full ISD dataset (Smith et al. 2011) that provides a more user-friendly format with a modified time stamp that corresponds to the nearest hour of actual observation. ISD-Lite contains hourly ground-based observations for various parameters, including temperature and relative humidity. To ensure consistency in station locations and a sufficiently long observation record for the required variables, we selected 13 stations from the northeastern United States. These stations, primarily located at airports, provide approximately 30 years of near-continuous observations (1991–2020) without major changes in location or elevation. Therefore, we consider them representative of the long-term weather conditions in their respective areas, serving as reliable, multi-decadal records with hourly time resolution.

The second dataset comes from the U.S. Climate Reference Network (USCRN), a high-quality monitoring network covering the contiguous U.S. (CONUS), Alaska, and Hawaii (Diamond et al. 2013). USCRN sites are designed for long-term stability, with minimal expected changes to their surroundings for 50 to 100 years, ensuring that they accurately represent regional climatic conditions. The USCRN datasets used herein include approximately 15 years of observations (2009–2024) at a 5-minute temporal resolution. To maintain consistency with the ISD-Lite format, we applied the same “capture window” method used in ISD-Lite (National Centers for Environmental Information 2006), extracting observations closest to the top of each hour to convert USCRN data to an hourly resolution. For this study, we selected 24 USCRN stations across CONUS.

Taken together, hourly temperature and relative humidity time series from the 13 northeastern U.S. ISD-lite stations and the 24 USCRN stations from other regions of the U.S. represent diverse bioclimatic zones and weather conditions, and thus are well-suited for the HI evaluations performed in this study. A list of the 37 stations, their latitude and longitude locations, elevations, and related information is provided in Supplementary Materials S1.

To explore the implications of our findings beyond the station data, we use the gridMET high-resolution (1/24th degree ~ 4 km) gridded surface meteorological data product (Abatzoglou 2013) across the contiguous U.S. for the 1979–2020 period. The gridMET temperature and humidity data is a hybrid product, in effect merging information from the high resolution PRISM gridded data (Daly et al. 2008) and the North American Land Data Assimilation System Phase 2 (Mitchell et al. 2004).

Because we are interested in studying days with high HI values, some analyses presented here focus on the months of May through September - a period commonly referred to as the heat season in the Northern Hemisphere midlatitudes. Other analyses in this work define extreme heat days as any day of the year when HI exceeds the 95th percentile of daily maximum HI (*hismax24*) values computed over the months of June, July, and August (JJA) for a multi-year reference period. The local JJA 95th percentile value of daily maximum HI has been shown by Hondula et al. (2022) to be the climatological indicator most strongly correlated with when NWS Weather Forecast Offices across the U.S. issue public heat alerts.

### Heat index calculations and comparisons

In this study, we use the adaptedheat.index function in the weathermetrics R package (Anderson et al. 2013b) to calculate HI. The algorithm duplicates what is used operationally by the NWS to compute HI using station-based temperature and relative humidity data, though it is not identical to the original Steadman (1979) method.

To facilitate calculations, Steadman’s (1979) results, which are based on a collection of equations representing multiple environmental states, were subsequently approximated via a single polynomial fit (Rothfusz 1990). Described by Rothfucz as “an ersatz version of the HI equation”, the polynomial fit was adopted for widespread use in the NWS, with some modest modifications added (Weather Prediction Center 2022), and continues to be part of NWS summer forecasts, including heat advisories and excessive heat watches and warnings (Hawkins et al. 2017). Anderson et al. (2013b) examined more than 20 different HI algorithms and concluded that the “NWS algorithm agreed best with Steadman’s apparent temperature by all metrics considered and for both of Steadman’s original tables.”

Lu and Romps (2022) noted that in extremely hot and humid conditions, Steadman’s thermoregulation model becomes unphysical, resulting in problematic values of the NWS HI. To ensure our results are not influenced by these invalid values, we calculated the percentage of heat season days when *hismax24* falls outside the Steadman model’s designed range, as shown in Supplemental Material S1. As a quality-control check, we find 25 stations have zero cases of out-of-range *hismax24* values, 11 have 0.51% or fewer such days, and only one station (Port Aransas, Texas) has a non-negligible number (4.7%). Given the overall rarity of days with temperature and humidity combinations exceeding Steadman’s valid range, we consider these station data fit for purpose.

Our objective is to compare two approaches for calculating the daily maximum HI: (1) the standard calculation workflow, which uses hourly station data, and (2) an approximation workflow, which relies on daily extreme temperature and relative humidity values.

**Fig. 1:**
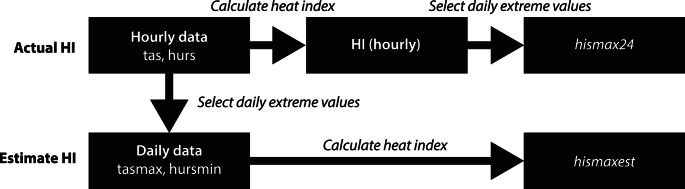
Schematic workflows depicting how *hismax24* and *hismaxest* are calculated. The standard calculation workflow (Actual HI) to calculate *hismax24* is shown in the top row and the approximation workflow (Estimate HI) to calculate *hismaxest* is shown in the bottom row

In the standard workflow, we first compute the hourly HI using hourly station temperature and relative humidity data. We then determine the daily maximum HI by selecting the highest value within each 24-hour period. This method provides a benchmark value for the daily maximum HI, which we refer to as *hismax24* (Fig. 1).

In the approximation workflow, designed to align with climate and earth system model outputs, such as those that are part of the Coupled Model Intercomparison Project (CMIP) (Eyring et al. 2016), we first extract the daily maximum temperature (*tasmax*) and daily minimum relative humidity (*hursmin*) from the hourly station data for each calendar day. We then compute the approximate daily maximum HI using *tasmax* and *hursmin* (Fig. 1). This method provides an estimated daily maximum HI value, which we refer to as *hismaxest*, that is similar to what could be obtained from climate and earth system model outputs that lack hourly data for these variables. For a given day, this *hismaxest* estimation can either exactly match *hismax24* (i.e., *hursmin* occurs at the same hour as *tasmax* and the daily maximum HI) or underestimate it (e.g., the relative humidity was greater than *hursmin* at the actual hour of maximum HI). This holds true when calculating HI using the original Steadman (1979) model, an extended HI method (Lu and Romps 2022), and the Rothfusz regression (1990) with several adjustments used in the US NWS HI algorithm. This conclusion presumes HI increases monotonically with temperature and relative humidity, which is applicable in all the above-mentioned calculations.

The rationale for the use of *tasmax* and *hursmin* to estimate the daily maximum HI assumes that the greatest heat stress is felt at the time of the highest near-surface air temperature. As temperatures increase, the amount of water vapor required for air to reach saturation increases, following the Clausius-Clapeyron equation. Thus, an inverse relationship exists between temperature and relative humidity. Assuming that the specific humidity (or vapor pressure or dew point) remains nearly constant during the day, it follows that the day’s lowest relative humidity would occur at the time of the maximum temperature, and at that time HI would be at its peak.

However, this approximation can fail in a number of ways. Weather conditions can cause specific humidity levels to markedly change during the day at a location, leading to the decoupling of *hursmin* and *tasmax*. In this case, *tasmax* could occur at the same time as *hismax24*, but not *hursmin*. Alternatively, *tasmax* and *hursmin* could occur at the same time, but there may be another combination of hourly *tas* and *hurs* that yields a higher *hismax24*. To take this into consideration, we identify the hour of occurrence for *hismax24*, *tasmax*, and *hursmin* during our analysis to elucidate the underlying cause of the underestimation.

Focusing on the time of year when extreme heat events occur, our comparison of *hismax24* and *hismaxest* follows two approaches:

1. Direct Comparison: During each day of the heat season (defined as May, June, July, August, and September, MJJAS), we calculate the difference between *hismaxest* and *hismax24* at each station. We determine how often *hismax24* and *hismaxest* match exactly (i.e., the fraction of days for which *hismaxest* is a perfect approximation of *hismax24*) and for days when *hismaxest* does not equal *hismax24*, we note the frequency with which the differences fall within specific degree Celsius and degree Fahrenheit ranges.
2. Threshold-Based Comparison: At each station, we define an extreme heat occurrence as any day of the year when the daily maximum HI exceeds the climatological 95th percentile of *hismax24* calculated over JJA. This threshold was examined by Hondula et al. (2022) to be the climatological indicator that most strongly correlated with heat alert warnings and heat advisories issued by the U.S. NWS. For each station, we count the number of extreme heat days exceeding this local threshold in each of the *hismax24* and *hismaxest* time series and compare the two calculation methods’ results, measuring how often *hismaxest* underestimates these events. Unlike the direct comparisons, the threshold-based comparisons focus on only the hottest days in the stations’ records.

### Modeling the underestimation of extreme heat index days

Further examining the stations with a high underestimation of extreme heat days from the threshold-based comparison, we investigate the relationship between local conditions and the underestimation ratio. We derive a two-part nonlinear model capturing the relationship between the HI 95% threshold at each station and the frequency of underestimation of extreme heat days. We then apply the model to threshold values derived from the gridMET high-resolution gridded reanalysis dataset (Abatzoglou 2013), as described in Supplementary Material S3. This produces a general estimate of spatial variability in underestimated extreme HI day counts across the contiguous U.S.

## Results

### Quantitative comparison between *hismax24* and *hismaxest*

**Fig. 2:**
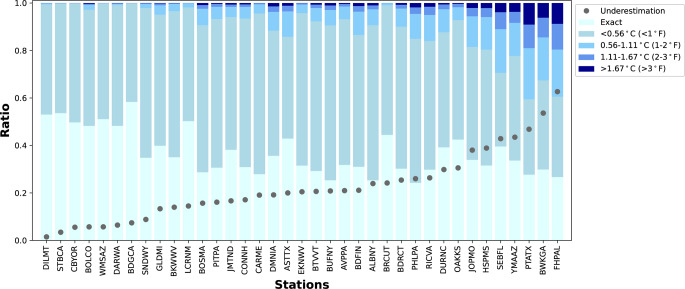
Station-wise comparison of *hismax24* and *hismaxest*. The bar plot shows the ratio of days during the heat season (MJJAS) when *hismaxest* is exact or lower than *hismax24*, binned by temperature difference ranges. The grey dots represent the underestimation of the number of extreme heat days (any day exceeding the local climatological JJA 95% percentile *hismax24*) calculated using *hismaxes*t compared to *hismax24*. Stations are ordered from the smallest to the largest underestimation of the fraction of extreme HI days. Additional information for individual stations, including identifier codes, can be found in Supplementary Material S1

The direct comparison of how well the daily *hismaxest* (calculated from daily *tasmax* and *hursmin*) estimated *hismax24* values varied dramatically across our 37 contiguous US stations. However, the direct comparison results indicate fairly good agreement between *hismax24* and *hismaxest* at the majority of our sites, both in the frequency of exact matches and in the magnitude of temperature differentials between the two methods (Fig. 2). At all stations, at least 60% of days have differences within 0.56 °C (1 °F), and 32 stations have more than 80% of days with less than 0.56 °C difference. Bodega, CA has the highest exact match rate, with 58% of days showing identical values, while Buffalo, NY and Philadelphia, PA have the lowest at 24%. Four USCRN stations in the western U.S.—Santa Barbara, CA; Coos Bay, OR; Williams, AZ; and Bodega, CA—show the smallest degree differences, with their largest discrepancies computed over 15 heat seasons remaining under 0.56 °C (1 °F). In contrast, 23 out of 37 stations have their largest differences exceeding 1.67 °C (3 °F). These direct comparison results suggest that, for some types of applications and locations, *hismaxest* may serve as a reasonable approximation of *hismax24*.

However, the threshold-based comparison, which focuses on the occurrence of days with extremely high HI values, reveals a less ideal performance. As expected, stations with more frequent large differences between *hismax24* and *hismaxest* also show greater underestimation of extreme heat days. This underestimation varies widely, ranging from as low as 2% in Dillon, MT, to as high as 63% in Fairhope, AL (Fig. 2). Overall, the *hismaxest* approximation misses more than 20% of the extreme heat days at roughly half of the 37 stations.

**Fig. 3:**
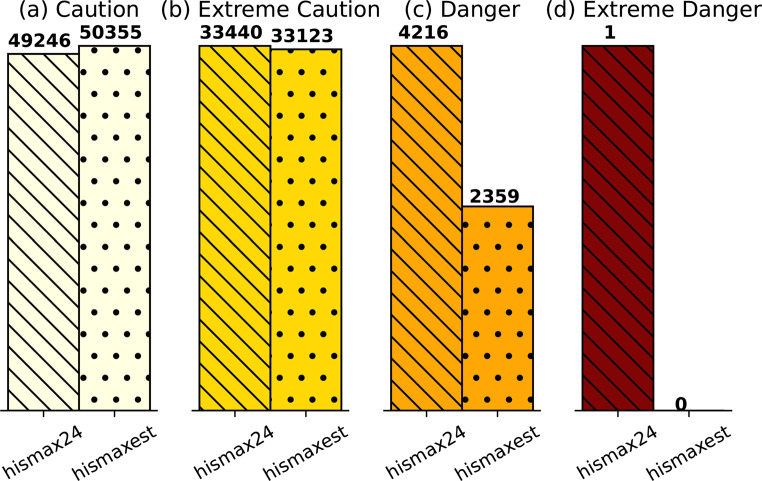
Total number of days falling within each U.S. National Weather Service (NWS) heat warning category, as calculated by *hismax24* and *hismaxest*, aggregated across all stations. The ‘Caution’ (**a**) category is defined as HI of 80°F–90°F, ‘Extreme Caution’ (**b**) as 90°F–103°F, ‘Danger’ (484_2025_3001) as 103°F–125°F, and ‘Extreme Danger’ (**d**) as above 125 °F. The exact day counts are displayed above each bar. Note that the y-axis scale varies between panels. A station-level breakdown is provided in Supplementary Material S2

To assess how the approximation affects the total number of days within each heat warning category defined by the U.S. NWS, we summed the number of days across all CONUS stations, as shown in Fig. 3. The U.S. NWS uses a threshold-based approach with the following categories: Caution (80°F ≤ HI<90°F), Extreme Caution (90°F ≤ HI<103°F), Danger (103°F ≤ HI<125°F), and Extreme Danger (125°F ≤ HI) (NWS 2025). The results indicate that *hismax24* and *hismaxest* have very similar day counts in the ‘Caution’ and ‘Extreme Caution’ categories. We note that *hismax24* has slightly fewer days in the ‘Caution’ category, because more days are classified into higher severity categories. In the ‘Danger’ category, *hismaxest* misses nearly 30% of the days identified by *hismax24*, and in the ‘Extreme Danger’ category, it fails to capture any days at all. These findings suggest that the underestimation becomes more significant with increasing heat severity.

### Modeling the underestimation of extreme HI days

**Fig. 4:**
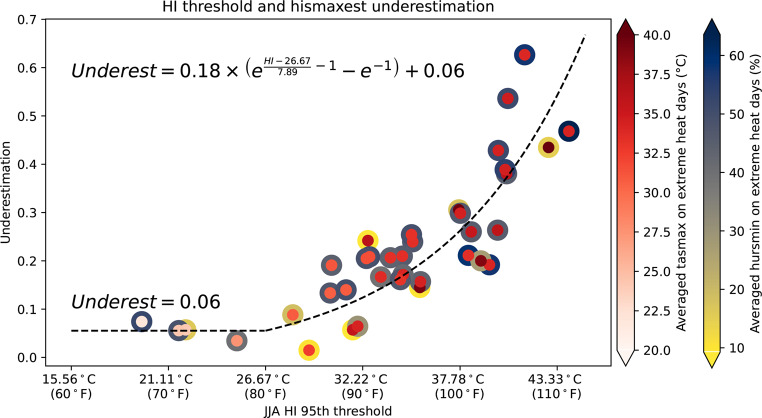
Relationship between the local climatological 95th percentile HI (°C/°F) during June–July–August (JJA) and the underestimation ratio of count of extreme heat days using *hismaxest* versus *hismax24*. Each dot represents a station. The color of the inner circles represents average *tasmax* on extreme heat days, and the color of the outer circles represents average *hursmin* on extreme heat days. The flat dashed line represents the underestimation regression model for HI < 26.67 °C (80 °F), and the curved dashed line represents the exponential fit regression for HI ≥ 26.67 °C (80 °F)

We examine the stations with a high underestimation of extreme HI days in Fig. 2 and find that these stations are primarily, but not exclusively, located in the hot and humid regions of the southern U.S. To visualize and investigate this relationship, the underestimation of extreme heat days is plotted against the HI JJA 95% threshold (Fig. 4) and the plot reveals a nonlinear relationship: stations with a higher HI threshold tend to have a greater underestimation of extreme heat days, and this underestimation increases nonlinearly as the HI threshold rises. To capture this pattern, we use a two-part nonlinear model.

Because the trend begins to increase noticeably when the HI threshold exceeds 26.67 °C (80 °F), and the NWS classifies HI > 80 °F as a caution for heat exposure (Maung and Tustin 2020; OSHA 2022), we divide the data into two groups at this threshold. For stations with an HI threshold below 26.67 °C (80 °F), underestimation remains relatively constant, therefore we use the average underestimation ratio as a fixed value (0.06) in the model (Fig. 4 flat dashed line). For stations with an HI threshold above 26.67 °C (80 °F), we fit an exponential model to describe the nonlinear increase, ensuring it intersects with the constant model at 26.67 °C (80 °F) (Fig. 4 curved dashed line). This simple model provides the estimation of a quantitative relationship between extreme heat day underestimation using *hismaxest* and local HI conditions, offering insights into the limitations of *hismaxest* in hot and humid environments.

To better understand how underestimation of extreme HI days relates to local temperature and humidity, color shading of the inner and outer portions of Fig. 4’s circles indicates the average tasmax and *hursmin*, respectively, on extreme heat days for each station. No clear pattern is evident to suggest that *tasmax* or *hursmin* alone dominates the underestimation. High HI values can arise from different combinations—either high *tasmax* with low *hursmin* or moderate *tasmax* with high *hursmin* (e.g., the two rightmost points). Both types of conditions show similar levels of extreme HI day count underestimation when using the *hismaxest* approximation, suggesting that it is the combined effect of temperature and humidity that matters most.

**Fig. 5:**
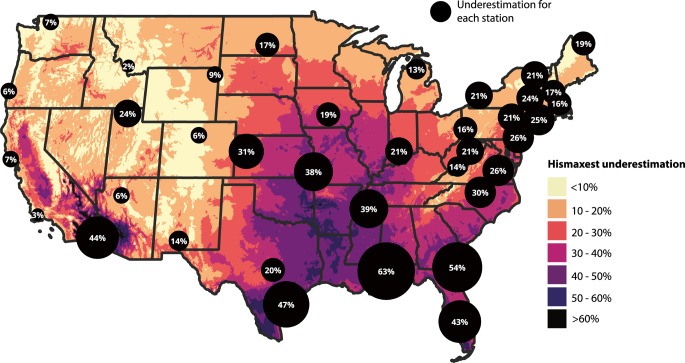
Spatial distribution of the underestimation of extreme HI day counts across the contiguous U.S. Black circles show underestimation of extreme HI days computed from station data, with circle size indicating magnitude. The CONUS-wide color shading approximates the underestimation as derived by applying the model from Fig. 4 to the adjusted 95% percentile JJA HI thresholds calculated from the high resolution (1/24° grid) gridMET *tasmax* and *hursmin* data (see supplementary material S3 for information on the adjustment applied). Note that the color shading is a rough approximation due to the variability observed in the underlying model (Fig. 4)

Applying the model depicted in Fig. 4 to the adjusted *hismaxest* values calculated from the gridMET *tasmax* and *hursmin* (Supplementary materials S3) yields a general estimate of geographic variations in the central tendency of extreme HI day count underestimations across the contiguous U.S. (Fig. 5 the color-shaded CONUS-wide background). As illustrated by the scatter around the fitted distribution in Fig. 4, undercount values at individual locations may vary noticeably from estimates derived from the fitted equations. Where the model-predicted underestimation ratio is generally below 10% (e.g., cool and/or dry regions, such as the Rocky Mountains, portions of the northeast and parts of the western third of CONUS), *hismaxest* can be expected to be a good approximation for *hismax24* during the heat season. In contrast, the model predicts the largest underestimation in the southern U.S., particularly in the deserts of California and Arizona, and parts of Alabama, Texas, Louisiana, Oklahoma, Arkansas and Florida, where underestimation can exceed 50%. Alarmingly, these regions are among the most vulnerable to future extreme heat events (Wobus et al. 2018). Relying on the *hismaxest* approximation when assessing extreme heat event frequency in these areas may severely underestimate risks, potentially contributing to inadequate heat preparedness and preventive measures.

We note that the color shading in Fig. 5 is not expected to perfectly match observed station-level data. Differences can arise due to gridMET grid cell values not being identical to those for a station within a given grid cell, and the imperfect fit depicted in Fig. 4. Thus, the color shading should be interpreted as a general depiction of geographic variations in biases introduced by the hismaxest approximation, rather than a basis for precise quantitative analysis.

## Discussion

This study is motivated by the desire to evaluate the effectiveness of a commonly used method for approximating the daily maximum heat index based on daily weather measurements - an approximation often employed when hourly or sub-hourly temperature and humidity data is unavailable. As can be inferred from results presented in Figs. 2, 3, 4 and 5, the most straightforward answer to whether *hismaxest* is a reliable approximation of hismax24 is “it depends”. Of particular concern are cases which might use the *hismax24* approach to determine the daily maximum HI from hourly station observations during a historical reference period and adopt another method, such as *hismaxest*, to estimate daily maximum HI using daily variable time series derived from climate and earth system model projections. Results here indicate experimental designs with this type of methodological inconsistency can introduce discontinuities in the results, leading to a marked underestimation of the projected future frequency of extreme HI days at some locations.

To further elucidate the cause of why *hismaxest* can be a poor estimation of *hismax24*, we highlight specific synoptic weather conditions that can cause the daily minimum relative humidity (*hursmin*) and maximum air temperature (*tasmax*) to occur at very different times than *hismax24*, challenging the assumption that they occur simultaneously and thus making *hismaxest* an imprecise approximation of *hismax24* on such days. We go on to describe why the *hismaxest* approximation tends to be more problematic in hotter regions and on extremely hot days, while performing better in cooler, drier circumstances.

As noted previously, Steadman’s (1979) physiology-based model is not applicable for some extreme temperature and humidity conditions. However computer code implementations of the NWS HI typically return values regardless of whether conditions are within the range considered by Steadman. When discussing our study’s results, we note the relatively rare occasions when out-of-range temperature and humidity combinations may have a small effect on quantitative results.

### Discrepancies between *hismaxest* and *hismax24*

**Fig. 6:**
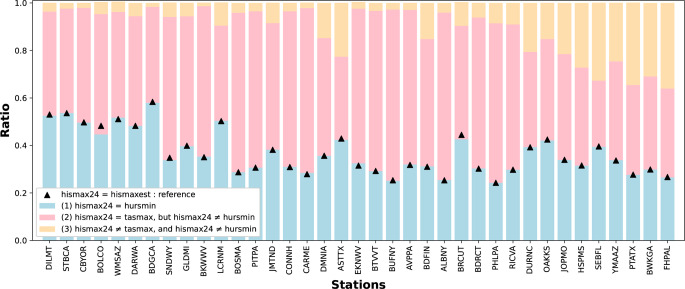
Breakdown of temporal conditions under which *hismaxest* is an exact match or an underestimate of *hismax24*. The triangle dots represent the ratio of MJJAS days where *hismaxest* is exactly equal to *hismax24*, corresponding to the “Exact ratio” in Fig. 2. The blue bars indicate the proportion of days when *hismax24* coincides with the time of *hursmin*. The pink bars show the proportion of days when *hismax24* occurs at the same time as *tasmax*, but differs from *hursmin*. The orange bars represent cases where *hismax24* does not occur at the same time as *tasmax* or *hursmin.* As in Fig. 2, stations are ordered from low to high underestimation of extreme HI days

For a first approximation we looked at how often *tasmax* and *hursmin* actually occur at the same time (Supplementary Material S4). We see that for all 37 stations this assumption holds true approximately 60% of the time. However, this does not line up with the exact match ratios seen in Fig. 2. To further investigate the discrepancies between *hismaxest* and *hismax24*, we examine the temporal conditions influencing their mismatch. Since *hismaxest* is derived using the daily maximum temperature (*tasmax*) and daily minimum relative humidity (*hursmin*), we identify three possible cases that describe when *hismax24* occurs (Fig. 6):

1. *hismax24* occurs at the same time as *hursmin*.
2. *hismax24* occurs at the same time as *tasmax*, but not at *hursmin*.
3. *hismax24* occurs at a different time from both *tasmax* and *hursmin*.

For situation (1), when *hismax24* occurs at the same time as *hursmin*, in most summer weather conditions, *hismaxest* is equal to *hismax24*. This consistency is evident in the comparison of the triangle dots and the blue bars in Fig. 6. This is because *hismax24* occurring at *hursmin* is a sufficient condition, but not a necessary condition for the approximation that *hismaxest* equals *hismax24*. When *hismax24* aligns with *hursmin*, the corresponding temperature can only be the highest temperature of the day. Under such conditions, *tasmax*, *hursmin*, and *hismax24* occur simultaneously, resulting in *hismaxest* being equal to *hismax24*.

However, there are a few exceptions at some stations. For example, we observe a mismatch in Boulder, CO, where *hismaxest* still equals *hismax24* even when *hismax24* does not occur at *hursmin*. A closer look at the records for this station reveals that on certain MJJAS days, the temperature never exceeds 4.44 °C (40 °F). Under such cool conditions, the NWS HI is set to be the same as the air temperature, regardless of relative humidity. Therefore, on those days, *hismaxest* always equals *hismax24*, which is also the same as *tasmax*.

For situation (2), when *hismax24* occurs at the same time as *tasmax*, but differs from *hursmin*, the conditions are such that at the time of peak temperature, the corresponding relative humidity is higher than the daily minimum humidity. As a result, *hismax24*, which is calculated using *tasmax* and this higher relative humidity, is greater than the *hismaxest* calculated from *tasmax* and *hursmin*.

**Fig. 7:**
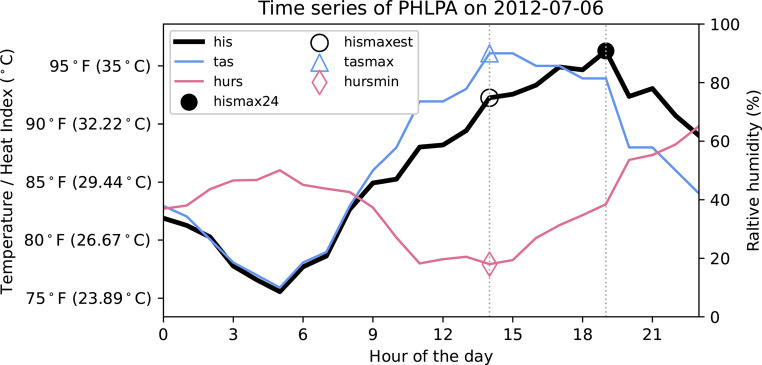
Case study of July 6th, 2012 at the PHLPA ISD-lite station when *tasmax*, *hursmin*, and therefore *hismaxest*, occur at a different time than *hismax24*. The pink line is the hourly *hurs* (%) time series with a diamond at *hursmin*, the blue line is the hourly *tas* (°C/°F) time series with a triangle at *tasmax*, and the black line is the hourly *his* (°C/°F) time series with an open circle at *hismaxest* and a closed circle at *hismax24*

For situation (3), *hismax24* does not occur at the same time as *tasmax* nor *hursmin*. We find an example of this in Philadelphia, PA, on July 6, 2012 (Fig. 7). On that day, even though *tasmax*, *hursmin*, and therefore *hismaxest*, occur at the same time in the afternoon at 14:00, it is not the moment that *hismax24* is at its peak. Due to a slow decrease in air temperature and a rapid increase in relative humidity, *hismax24* occurs later in the evening at 19:00. Because of the strong influence of relative humidity, temperature no longer dominates the HI trend, leading to different peak times for *tasmax* and *hismax24*, and an underevaluation for *hismaxest* compared to *hismax24*.

Figure 6 also shows the ratio of days in which these situations occur across different stations. Consistent with Fig. 2, at stations with greater underestimation, there are fewer days where *hismax24* coincides with *hursmin*, leading to more cases where *hismaxest* is not equal to *hismax24*. Comparing stations with the largest underestimation of extreme hot days (right) to those with the smallest (left) reveals a clear pattern: stations with the largest underestimations exhibit a greater frequency of case 3 (*hismax24* not simultaneous with *tasmax*) and a similar ratio of case 2. This difference highlights the increasing frequency of case 3 as the likely cause of the more substantial underestimation at stations for which *hismaxest* performs poorly, indicating that *tasmax* is no longer the primary contributing factor of *hismax24* at those sites. These results suggest that relative humidity plays a more important role in regulating the diurnal cycle of *hismax24* at hotter stations with higher 95% thresholds, which are also the stations with greater underestimations of extreme heat days.

### The sensitivity of the heat index to relative humidity varies with temperature

**Fig. 8:**
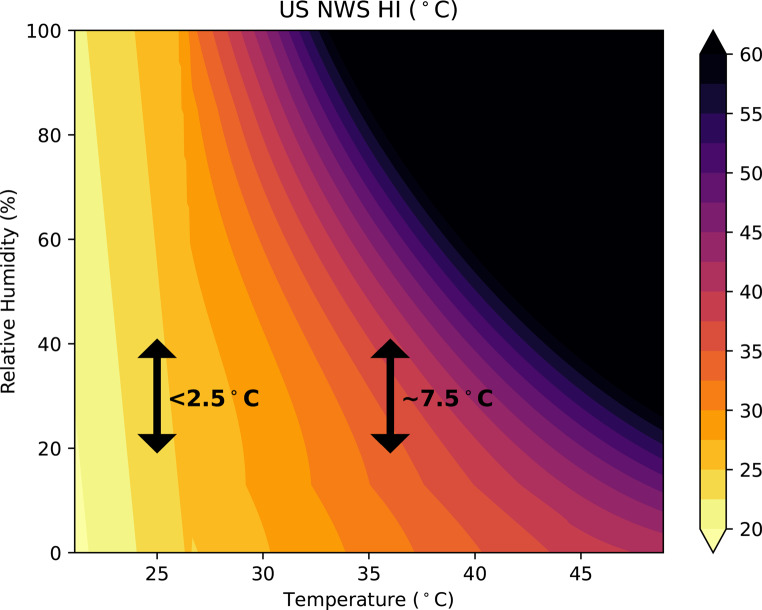
HI (°C) contour plot derived from temperature (°C) and relative humidity (%), with 2.5 °C as the color interval. The black arrows indicate a change in relative humidity from 20–40% and the corresponding change in HI°C when temperature is held constant at 25–36 °C

The annotated heat index chart in Fig. 8 illustrates how the sensitivity of the heat index to changes in relative humidity varies with temperature. One can infer the relative roles of temperature and relative humidity from the orientation of the HI contours, with humidity’s role increasing at higher temperatures where contours become less vertical. For example, when the temperature is 25 °C, an increase in relative humidity from 20 to 40% leads to a HI increase of less than 2.5 °C. However, when the temperature exceeds 36 °C, the same relative humidity change results in a 7.5 °C increase in the HI.

As noted in Simpson et al. (2023), the sensitivity of commonly used TSIs to humidity differs across indices and across temperature and humidity regimes. Whereas all TSIs were found to be in rough agreement regarding the importance of humidity under hot and humid conditions, the NWS HI was identified by Simpson et al. (2023) as being among the TSIs that are less sensitive to humidity in the hot-dry regime.

The following discussion offers a simplified summary of the human thermoregulatory model developed by Steadman (1979), highlighting key factors pertaining to why estimates of daily maximum HI values calculated based on daily maximum temperature and minimum relative humidity (*hismaxest*), are more prone to significantly underestimate the true daily maximum HI (*hismax24*) on extremely hot days. For more complete details about Steadman’s multi-faceted methodology, upon which the NWS HI is based, the interested reader may refer to Steadman (1979) and Lu and Romps (2022).

Steadman’s model is intended to be applicable to an idealized and adaptable healthy adult human experiencing a light breeze of 2.5 m s^−1^ in the shade. A default metabolic heat production rate consistent with walking at a speed of 1.4 m s^−1^ is presumed. Overall, the model seeks to maintain a constant body core temperature of 37 °C and core vapor pressure at 5.65 kPa across different ranges of air temperature and humidities. Thus, thermal equilibrium is achieved as the heat generated within the body equals the amount lost via exhaled air and from the skin surface. At the base state, specific humidity is also fixed to be a reference level at 1.6 kPa (dewpoint of 14.0 °C or 57.2 °F), and the only to-be-determined variable is temperature. A person is assumed to have the same body feeling under the above base state, as in the real-world environment with actual temperature and specific humidity combination. In the real-world environment, if it is more humid (a change in specific humidity), people perceive it as feeling hotter (a change in temperature) because of the reduced evaporative cooling, and the model translates the feeling induced by moisture variation to an ‘apparent’ or ‘feels like’ temperature.

The model also includes adaptive assumptions, such as reducing the amount of clothing worn under ‘sultry’ conditions. We find that 34 of the 37 stations studied have more than 95% of their extreme heat days (*hismax24* > JJA 95th percentile values) lie in Steadman model’s ‘unclothed’ regime (HI > ~ 298 K/76.7°F/24.85°C), where sweating is active and clothing resistance is ignored. Excluding three cooler exceptions (Bodega California, Boulder, Colorado, and Coos Bay, Oregon) that have small underestimations of extreme heat days from *hismaxest*, our discussion focuses on the ‘unclothed’ regime.

In such hot conditions in the ‘unclothed’ regime, the HI is more sensitive to the same percentage change in relative humidity (Fig. 8) than under cooler conditions. This nonlinearity can be explained by the combination of physical science and physiological response factors. From the physical science standpoint, we note that Steadman’s model simulates the apparent temperature using specific humidity, not relative humidity. As the saturated vapor pressure increases nonlinearly with temperature, the same percentage change in relative humidity coincides with a larger change in specific humidity at higher temperatures, which drives a larger change in the corresponding apparent temperature. From the physiological standpoint, the human body responds to high temperatures through vasodilation and sweating. Vasodilation supports radiative and sensible heat exchange by increasing blood flow near the skin, while sweating provides a more efficient way to release heat through the evaporation of moisture. When under increased heat stress conditions, the body sweats more as a means to enhance the evaporative cooling rate and this cooling becomes more efficient at higher temperatures. However, its effectiveness strongly depends on the humidity of the surrounding environment. When humidity rises, it becomes harder for sweat to evaporate, making it more difficult for the body to cool down. As a result, the body perceives the environment as much hotter than it actually is, reflected in a higher apparent temperature.

## Summary

This study evaluates the uncertainty of a commonly used approximation for estimating the daily maximum heat index (*hismax24*) using station-based hourly temperature and relative humidity observations. We compare the HI calculated from the approximation method (*hismaxest*), which estimates HI from daily maximum temperature and minimum humidity values, against the standard calculation (*hismax24*), which is the daily maximum of the HI calculated each hour of the day. Analyses using long-term datasets from NOAA’s Integrated Surface Database (ISD-Lite) and the U.S. Climate Reference Network (USCRN) reveal that while *hismaxest* performs reasonably well in absolute difference comparisons—showing deviations of less than 0.56 °C (1 °F) at least 60% of the time—significant underestimation occurs when detecting extreme heat events defined by a threshold. The threshold-based comparisons performed at 37 U.S. stations reveal that using *hismaxest* can result in as little as a 2% underestimation, but up to a 63% underestimation, in the number of extreme heat days.

A key finding is the nonlinear relationship between underestimation of the frequency of extreme HI days and local HI thresholds, where regions with higher thresholds experience greater underestimation. Applying a two-part model across the contiguous U.S., we confirm that the largest discrepancies occur in hotter regions where underestimation can exceed 50%. Two contributing factors of the underestimation are the *hismaxest* assumptions that *tasmax* and *hursmin* occur simultaneously and *hismax24* also occurs at that time - assumptions that can be violated, particularly in hotter regions. At higher temperatures, HI becomes increasingly sensitive to humidity due to the nonlinear relationship between temperature and moisture transfer in the human thermoregulatory model. This increasing sensitivity leads to larger discrepancies between *hismaxest* and *hismax24*, resulting in a greater underestimation of extreme hot days in hotter regions.

This study brings quantitative insight into whether the commonly used daily estimate, *hismaxest*, can reliably approximate *hismax24*. Our findings indicate that its accuracy varies with the location of interest. In cool and dry climates, using *tasmax* and *hursmin* can provide a reasonable approximation. For example, Diaconescu et al. (2023) find that *tasmax* and *hursmin* can provide a good estimate for the humidex - a heat-stress index used by the Meterological Services in Canada, which is broadly similar to the US NWS HI in that it is a function of near-surface temperature and humidity. However, our results show that in the hotter southern regions of the US, this daily approximation can significantly underestimate HI values, with more than 50% of extreme heat days missing in a threshold-based comparison at some locations and more than one-third missing over wider areas. As global climate change continues, the underestimation associated with the *hismaxest* approximation is expected to become more severe and affect a broader range of regions, as more areas experience higher temperatures. Additionally, as described by Lu and Romps (2022), the need to consider alternatives to the current NWS algorithm for computing the HI increases as high temperatures become more frequent.

The magnitude of underestimation of extreme heat index days from daily data found here can sometimes be as large as the inherent uncertainties associated with climate model sensitivities and emission scenarios (Intergovernmental Panel On Climate Change 2023). For example, Dahl et al. (2019) show that projected person-days per year with HI values above 100 °F have model ensemble uncertainties generally under 30%, and differences between high and low emission scenarios below 40%, across U.S. regions by mid-century. In contrast, our analysis indicates that the uncertainty introduced by the *hismaxest* approximation can reach approximately 30% for the ‘Danger’ category (103 °F threshold).

This underestimation highlights the value for CMIP GCMs and regional dynamical climate models to archive hourly data for a relatively small set of key surface variables that would enable more precise climate impacts analyses of interest to multiple disciplines. The availability of high temporal resolution model outputs for use as input to climate impacts studies that depend on capturing daily extremes across multiple weather variables can help reduce the risk of potentially misleading or contradictory conclusions. Because the importance of daily extrema of multivariate indices is not limited to thermal stress indices, our findings may be broadly applicable to a wider range of interdisciplinary research seeking to link combinations of meteorological conditions to climate impacts.

Our goal is not to discredit the current climate model-based heat projections or propose specific alternative analytical methods, but to highlight an underappreciated source of uncertainties in model-projected heat indices, as well as advocate for the value of more comprehensive hourly data availability. Faced with suboptimal climate model (Abatzoglou 2013) data availability, researchers’ desire to not “allow perfect become the enemy of the good” understandably leads to the use of imperfect, but generally assumed to be serviceable, approximations. Acknowledging the limitations of one common way to estimate daily maximum HI values, combined with prospects to expand data coverage, can help researchers refine their analyses, improve methodological consistency, and strengthen policy decisions for heat risk adaptation and mitigation.

**Table S1:** List of 37 weather stations used in our study. Note that temperatures at both ISD-lite and USCRN stations are recorded in increments of 0.1 K. Dew point temperatures are recorded at ISD-lite stations in increments of 0.1 K and subsequently converted to floating point relative humidity percentages. Relative humidity for USCRN stations is recorded as an integer percentage

	Station code	City	State	Data source	Latitude [°*N*]	Longitude [°W]	Elevation [m]	Missing days count	Missing days percentage	hismax24 threshold [°F]	hismaxest underestimation of extreme days [%]	Out of range hismax24 MJJAS days [%]
1	ALBNY	Albany	NY	ISD-lite	42.7	73.8	85.4	295	2.70%	95.1	23.90%	0
2	AVPPA	Pittston	PA	ISD-lite	41.3	75.7	289.9	236	2.20%	94.1	21.00%	0
3	BDRCT	Stratford	CT	ISD-lite	41.2	73.1	1.8	2212	20.20%	95	25.40%	0
4	BKWWV	Beaver	WV	ISD-lite	37.8	81.1	760.2	603	5.50%	88.3	14.00%	0
5	BOSMA	Boston	MA	ISD-lite	42.4	71	3.3	109	1.00%	95.9	15.70%	0
6	BTVVT	Burlington	VT	ISD-lite	44.5	73.2	101.1	117	1.10%	92.8	20.60%	0
7	BUFNY	Buffalo	NY	ISD-lite	42.9	78.7	216.2	2235	20.40%	90.7	20.80%	0
8	CARME	Caribou	ME	ISD-lite	46.9	68	188.6	2206	20.10%	86.8	19.10%	0
9	CONNH	Concord	NH	ISD-lite	43.2	71.5	103.2	247	2.30%	94.1	17.10%	0
10	EKNWV	Elkins	WV	ISD-lite	38.9	79.9	595.7	2287	20.90%	90.4	20.50%	0
11	PHLPA	Philadelphia	PA	ISD-lite	39.9	75.2	2.2	82	0.70%	101.2	26.00%	0.13%
12	PITPA	Pittsburgh	PA	ISD-lite	40.5	80.2	341	34	0.30%	93.9	16.10%	0
13	RICVA	Richmond	VA	ISD-lite	37.5	77.3	50.7	65	0.60%	103.9	26.40%	0.02%
14	ASTTX	Austin	TX	USCRN	30.6	98.1	136.1	886	15.20%	102.1	20.00%	0
15	BDFIN	Bedford	IN	USCRN	38.9	86.6	76	304	5.20%	100.9	21.10%	0.21%
16	BDGCA	Bodega	CA	USCRN	38.3	123.1	6.3	1187	20.30%	67.2	7.40%	0
17	BOLCO	Boulder	CO	USCRN	40	105.5	982.8	711	12.20%	71.8	5.70%	0
18	BRCUT	Brigham City	UT	USCRN	41.6	112.5	495.1	1011	17.30%	90.5	24.20%	0
19	BWKGA	Brunswick	GA	USCRN	30.8	81.5	2.5	443	7.60%	104.9	53.60%	0.18%
20	CBYOR	Coos Bay	OR	USCRN	43.3	124.3	1.2	529	9.10%	71.1	5.60%	0
21	DARWA	Darrington	WA	USCRN	48.5	121.4	40.7	1026	17.60%	89.5	6.50%	0
22	DILMT	Dillon	MT	USCRN	45.2	113	597.1	741	12.70%	84.5	1.50%	0
23	DMNIA	Des Moines	IA	USCRN	41.6	93.3	92.1	307	5.30%	103	19.20%	0.35%
24	DURNC	Durham	NC	USCRN	36	79.1	56.2	725	12.40%	100	29.90%	0
25	FHPAL	Fairhope	AL	USCRN	30.5	87.9	9.5	272	4.70%	106.7	62.70%	0.51%
26	GLDMI	Gaylord	MI	USCRN	44.9	84.7	146.1	721	12.30%	86.7	13.30%	0
27	HSPMS	Holly Springs	MS	USCRN	34.8	89.4	48.4	269	4.60%	104.6	38.90%	0.13%
28	JMTND	Jamestown	ND	USCRN	46.8	99.5	192	721	12.30%	91.8	16.70%	0.05%
29	JOPMO	Joplin	MO	USCRN	37.4	94.6	95.2	304	5.20%	104.8	38.00%	0.04%
30	LCRNM	Las Cruces	NM	USCRN	32.6	106.7	432.7	530	9.10%	95.9	14.50%	0
31	OAKKS	Oakley	KS	USCRN	38.9	101	287	377	6.50%	99.9	30.60%	0.05%
32	PTATX	Port Aransas	TX	USCRN	28.3	96.8	1.5	677	11.60%	111.2	46.80%	4.68%
33	SEBFL	Sebring	FL	USCRN	27.2	81.4	15	1039	17.80%	103.9	42.90%	0.05%
34	SNDWY	Sundance	WY	USCRN	44.5	104.4	579.2	756	12.90%	82.8	8.80%	0
35	STBCA	Santa Barbara	CA	USCRN	34.4	119.9	1.8	889	15.20%	77	3.40%	0
36	WMSAZ	Williams	AZ	USCRN	35.8	112.3	599	561	9.60%	89	5.70%	0
37	YMAAZ	Yuma	AZ	USCRN	32.8	114.2	62	480	8.20%	109.1	43.50%	0.04%

S2. Number of heat days within US NWS categories

## Electronic Supplementary Material

Below is the link to the electronic supplementary material.

Supplementary Material 1
PDF (513 KB)

## Data Availability

All heat index calculations were performed using the heat index function found in the R-language weathermetrics package version 1.2.2 (Anderson et al. 2013a, b) available at https://github.com/geanders/weathermetrics/. ISD-lite station data files and supporting information were obtained in February 2023 from a public data server maintained by NOAA’s NCEI https://www.ncei.noaa.gov/pub/data/noaa/isd-lite/. USCRN station data files and related information were downloaded in October 2024 from a public data server maintained by NOAA’s NCEI https://www.ncei.noaa.gov/pub/data/uscrn/products/subhourly01/.
