# Supplementary material for: Sometimes missing the heat: the risk of underestimating extreme heat days with daily maximum heat index approximation
Source: Int J Biometeorol. 2025 Aug 12;69(11):2967–81. doi: 10.1007/s00484-025-03001-7 (PMC12540600; doi:10.1007/s00484-025-03001-7)
Supplement: Supplementary file 1 — Supplementary Material 1 PDF (513 KB) [file 484_2025_3001_MOESM1_ESM.pdf]

Supplemental material for 'Sometimes Missing the Heat: The Risk of Underestimating Extreme Heat Days with Daily Maximum Heat Index Approximation':

S1. Summary table of station data

	Station code	City	State	Data source	Latitude [°N]	Longitude [°W]	Elevation [m]	Missing days count	Missing days percentage	hismax24 threshold [°F]	hismaxest underestimation of extreme days [%]	Out of range hismax24 MJJAS days [%]
1	ALBNY	Albany	NY	ISD-lite	42.7	73.8	85.4	295	2.70%	95.1	23.90%	0
2	AVPPA	Pittston	PA	ISD-lite	41.3	75.7	289.9	236	2.20%	94.1	21.00%	0
3	BDRCT	Stratford	CT	ISD-lite	41.2	73.1	1.8	2212	20.20%	95	25.40%	0
4	BKWWV	Beaver	WV	ISD-lite	37.8	81.1	760.2	603	5.50%	88.3	14.00%	0
5	BOSMA	Boston	MA	ISD-lite	42.4	71	3.3	109	1.00%	95.9	15.70%	0
6	BTVVT	Burlington	VT	ISD-lite	44.5	73.2	101.1	117	1.10%	92.8	20.60%	0
7	BUFNY	Buffalo	NY	ISD-lite	42.9	78.7	216.2	2235	20.40%	90.7	20.80%	0
8	CARME	Caribou	ME	ISD-lite	46.9	68	188.6	2206	20.10%	86.8	19.10%	0
9	CONNH	Concord	NH	ISD-lite	43.2	71.5	103.2	247	2.30%	94.1	17.10%	0

10	EKNWV	Elkins	WV	ISD-lite	38.9	79.9	595.7	2287	20.90%	90.4	20.50%	0
11	PHLPA	Philadelphia	PA	ISD-lite	39.9	75.2	2.2	82	0.70%	101.2	26.00%	0.13%
12	PITPA	Pittsburgh	PA	ISD-lite	40.5	80.2	341	34	0.30%	93.9	16.10%	0
13	RICVA	Richmond	VA	ISD-lite	37.5	77.3	50.7	65	0.60%	103.9	26.40%	0.02%
14	ASTTX	Austin	TX	USCRN	30.6	98.1	136.1	886	15.20%	102.1	20.00%	0
15	BDFIN	Bedford	IN	USCRN	38.9	86.6	76	304	5.20%	100.9	21.10%	0.21%
16	BDGCA	Bodega	CA	USCRN	38.3	123.1	6.3	1187	20.30%	67.2	7.40%	0
17	BOLCO	Boulder	CO	USCRN	40	105.5	982.8	711	12.20%	71.8	5.70%	0
18	BRCUT	Brigham City	UT	USCRN	41.6	112.5	495.1	1011	17.30%	90.5	24.20%	0
19	BWKGA	Brunswick	GA	USCRN	30.8	81.5	2.5	443	7.60%	104.9	53.60%	0.18%
20	CBYOR	Coos Bay	OR	USCRN	43.3	124.3	1.2	529	9.10%	71.1	5.60%	0
21	DARWA	Darrington	WA	USCRN	48.5	121.4	40.7	1026	17.60%	89.5	6.50%	0
22	DILMT	Dillon	MT	USCRN	45.2	113	597.1	741	12.70%	84.5	1.50%	0
23	DMNIA	Des Moines	IA	USCRN	41.6	93.3	92.1	307	5.30%	103	19.20%	0.35%

24	DURNC	Durham	NC	USCRN	36	79.1	56.2	725	12.40%	100	29.90%	0
25	FHPAL	Fairhope	AL	USCRN	30.5	87.9	9.5	272	4.70%	106.7	62.70%	0.51%
26	GLDMI	Gaylord	MI	USCRN	44.9	84.7	146.1	721	12.30%	86.7	13.30%	0
27	HSPMS	Holly Springs	MS	USCRN	34.8	89.4	48.4	269	4.60%	104.6	38.90%	0.13%
28	JMTND	Jamestown	ND	USCRN	46.8	99.5	192	721	12.30%	91.8	16.70%	0.05%
29	JOPMO	Joplin	MO	USCRN	37.4	94.6	95.2	304	5.20%	104.8	38.00%	0.04%
30	LCRNM	Las Cruces	NM	USCRN	32.6	106.7	432.7	530	9.10%	95.9	14.50%	0
31	OAKKS	Oakley	KS	USCRN	38.9	101	287	377	6.50%	99.9	30.60%	0.05%
32	PTATX	Port Aransas	TX	USCRN	28.3	96.8	1.5	677	11.60%	111.2	46.80%	4.68%
33	SEBFL	Sebring	FL	USCRN	27.2	81.4	15	1039	17.80%	103.9	42.90%	0.05%
34	SNDWY	Sundance	WY	USCRN	44.5	104.4	579.2	756	12.90%	82.8	8.80%	0
35	STBCA	Santa Barbara	CA	USCRN	34.4	119.9	1.8	889	15.20%	77	3.40%	0
36	WMSAZ	Williams	AZ	USCRN	35.8	112.3	599	561	9.60%	89	5.70%	0
37	YMAAZ	Yuma	AZ	USCRN	32.8	114.2	62	480	8.20%	109.1	43.50%	0.04%

Table S1 List of 37 weather stations used in our study. Note that temperatures at both ISD-lite and USCRN stations are recorded in increments of 0.1K. Dew point temperatures are recorded at ISD-lite stations in increments of 0.1K and subsequently converted to floating point relative humidity percentages. Relative humidity for USCRN stations is recorded as an integer percentage

S2. Number of heat days within US NWS categories

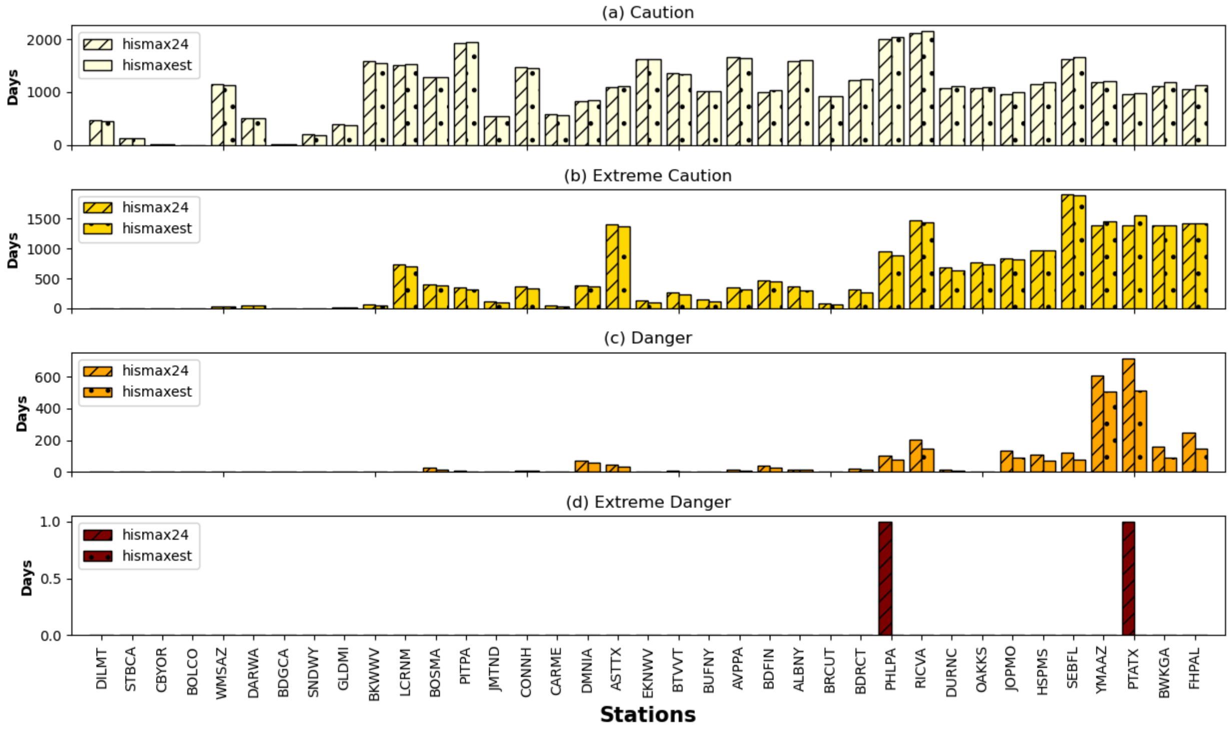

Fig. S2 Number of days within the four US NWS heat advisory categories, as calculated by *hismax24* and *hismaxest*, in 13 ISD-lite stations and 24 USCRN stations. The 'Caution' (a) category is defined as HI of 80°F–90°F, 'Extreme Caution' (b) as 90°F–103°F, 'Danger' (c) as 103°F–125°F, and 'Extreme Danger' (d) as above 125°F. Note that the y-axis of each subplot is different

S3. Adjustment function for 95th *hismax24* threshold with 95th *hismaxest* threshold

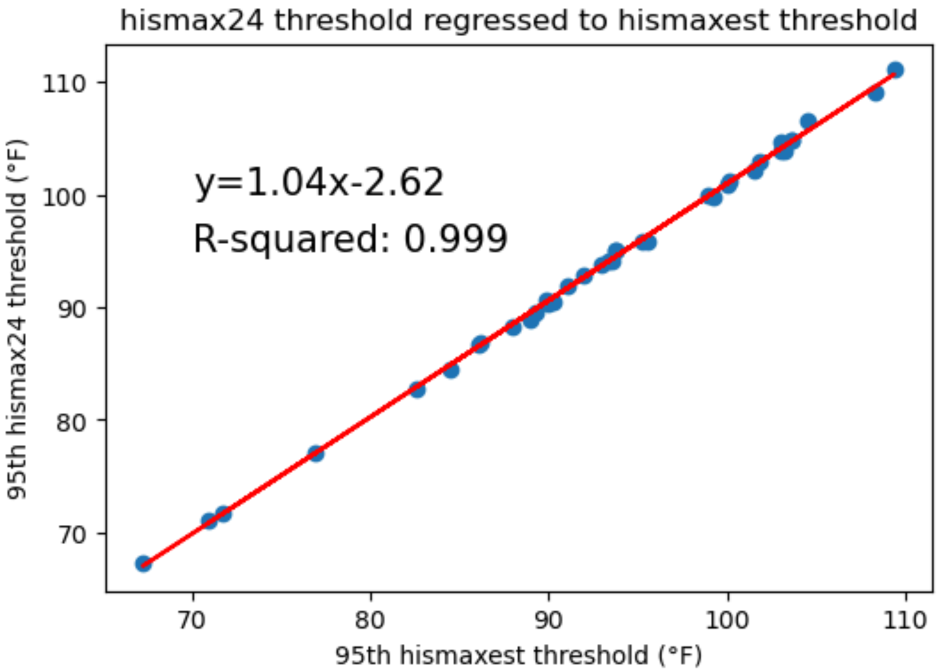

Fig. S3 Linear regression between the JJA 95th percentile *hismax24* and *hismaxest* thresholds across 37 stations. The regression equation and R-squared value are displayed on the plot

We applied this regression equation (JJA 95th *hismax24* ~ JJA 95th *hismaxest*; y=1.04x-2.62) to the JJA 95th percentile of *hismaxest* across the CONUS, calculated from gridMET *tasmax* and *hursmin* data. The resulting predicted 95th percentile *hismax24* values were then used in the equations from Figure 4 to estimate the expected underestimation of extreme heat days arising from the *hismaxest* approximation. These underestimations are shown as color contours in Figure 5.

Adjustments to JJA 95th *hismaxest* values add roughly 0.5°F to 1.5°F over the temperature range of interest, with the amount of adjustment increasing with higher thresholds (e.g., 80.0°F adjusts to 80.58°F; 90.0°F to 90.98°F; 100.0°F to 101.38°F).

We use the *hismaxest* threshold derived from gridMET, combined with the linear adjustment, because gridMET offers high spatial resolution (~4 km, 1/24°) and provides *tasmax* and *hursmin* data with validated data quality, supported by a dense weather station network (Abatzoglou 2013). By incorporating the color contours of underestimation ratios, our analysis offers a more continuous and spatially coherent visualization of the potential errors associated with using *hismaxest* to evaluate extreme heat days.

S4. Temporal ratio for *tasmax* coincides with *hursmin*

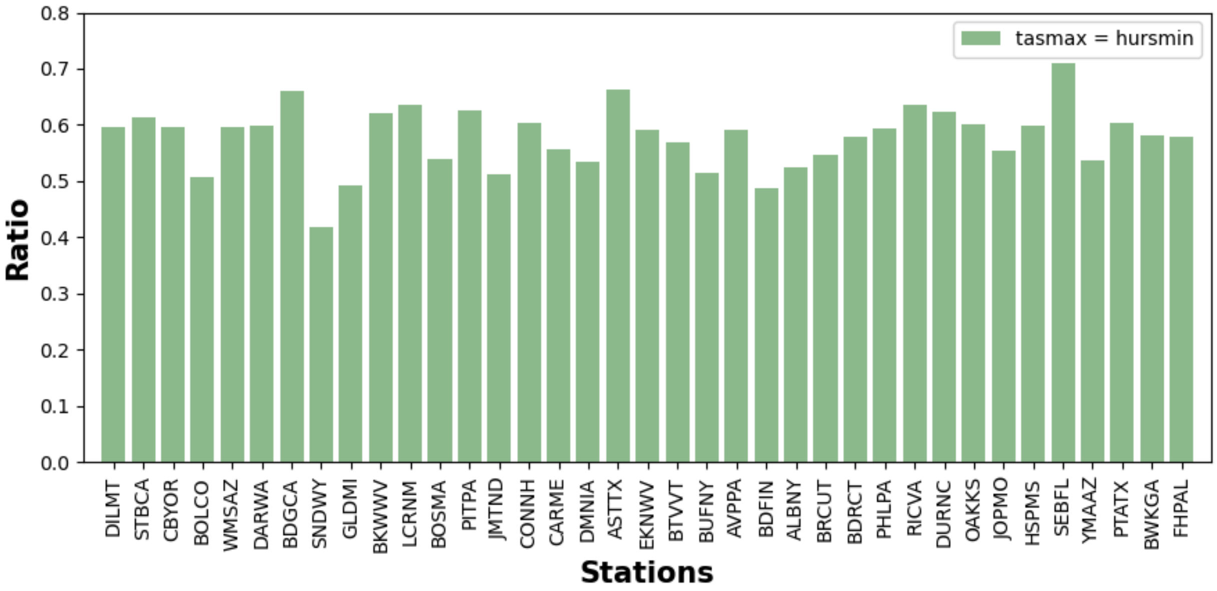

Fig. S4 The bar plot shows the ratio of days during the heat season (MJJAS) when *tasmax* and *hursmin* occur at the same time at 13 ISD-lite stations and 24 USCRN stations
